# Characterizing the microbiome of patients with myeloproliferative neoplasms during a Mediterranean diet intervention

**DOI:** 10.1128/mbio.02308-23

**Published:** 2023-10-25

**Authors:** Julio Avelar-Barragan, Laura F. Mendez Luque, Jenny Nguyen, Hellen Nguyen, Andrew O. Odegaard, Angela G. Fleischman, Katrine L. Whiteson

**Affiliations:** 1 Department of Molecular Biology and Biochemistry, University of California Irvine, Irvine, California, USA; 2 Department of Biological Chemistry, University of California Irvine, Irvine, California, USA; 3 Division of Hematology/Oncology, University of California Irvine, Irvine, California, USA; 4 Department of Epidemiology and Biostatistics, University of California Irvine, Irvine, California, USA; University of Hawaii at Manoa, Honolulu, Hawaii, USA; University Medical Center Groningen and University of Groningen, Groningen, the Netherlands

**Keywords:** gut microbiome, diet, myeloproliferative, neoplasm, Mediterranean

## Abstract

Myeloproliferative neoplasms (MPNs) are a class of rare hematological malignancies that result in the overproduction of myeloid lineage cells. These malignancies result in increased cytokine production and inflammation, which correlate with worsened symptom burden and prognosis. Other than bone marrow transplantation, there is no cure for myeloproliferative neoplasms. As such, treatments focus on reducing thrombotic risk, inflammation, and symptom burden. Because current pharmacological treatments carry significant side effects, there is a need to explore low-risk therapies that may modulate inflammation and alleviate symptom burden. One potential way to achieve this is adherence to a Mediterranean (MED) diet, which is rich in anti-inflammatory foods, reduces inflammatory biomarkers, and beneficially alters the gut microbiome. We performed a 15-week clinical trial of 28 individuals with MPN who were randomized to dietary counseling based on either a Mediterranean diet or standard U.S. Guidelines for Americans. Our primary objective was to determine whether MPN patients could adopt a Mediterranean eating pattern when supported with dietician counseling. As exploratory endpoints, we investigated the impact of diet and inflammation on the gut microbiome. Using shotgun metagenomic sequencing, we found that microbiome diversity and composition were stable throughout the study duration in both cohorts. Furthermore, we discovered significant differences in the microbiomes between MPN subtypes, such as increased beta-dispersion in subjects with myelofibrosis. Lastly, we found several significant correlations between the abundance of multiple bacterial taxa and cytokine levels. Together, this study provides insight into the interaction between diet, inflammation, and the gut microbiome.

**IMPORTANCE:**

The gut microbiome serves as an interface between the host and the diet. Diet and the gut microbiome both play important roles in managing inflammation, which is a key aspect of myeloproliferative neoplasm (MPN). Studies have shown that a Mediterranean (MED) diet can reduce inflammation. Therefore, we longitudinally characterized the gut microbiomes of MPN patients in response to Mediterranean or standard 2020 US Guidelines for Americans dietary counseling to determine whether there were microbiome-associated changes in inflammation. We did not find significant changes in the gut microbiome associated with diet, but we did find several associations with inflammation. This research paves the way for future studies by identifying potential mechanistic targets implicated in inflammation within the MPN gut microbiome.

## INTRODUCTION

Myeloproliferative neoplasms (MPNs) are a group of hematological malignancies defined by somatic mutations that activate JAK/STAT signaling in hematopoietic stem cells (1, 2). This results in an overproduction of myeloid lineage cells. Clinically, MPNs are divided into three clinical phenotypes: polycythemia vera (PV), essential thrombocythemia (ET), and myelofibrosis (MF). PV is characterized by an elevated red blood cell mass. Elevations in platelets and white blood cells are also common. Subjects with ET have elevated platelets but rarely have increased red or white blood cells. MF is characterized by reticulin fibrosis in the bone marrow, and often cytopenia. MF can develop from a “burn out” phase following PV or ET, termed post-PV or post-ET MF, or without a preceding diagnosis of PV or ET, termed primary myelofibrosis.

One feature of MPN is increased inflammatory cytokine abundance, which correlates with worsened symptom burden and disease prognosis (3, 4). MPN symptom burden can be severe, and many individuals experience fatigue, early satiety, abdominal discomfort, night sweats, pruritus, bone pain, fever, and unintentional weight loss. Other than bone marrow transplantation, there is no cure for MPN, and management focuses on reducing thrombotic risk and alleviating symptom burden. Current pharmacological treatments for MPN include JAK inhibitors, such as ruxolitinib, but these often carry significant side effects, like immunosuppression, weight gain, and increased skin cancer (5). Consequently, there is a need to explore low-risk alternatives for MPN management.

One method to non-pharmacologically manage MPN is through the consumption of a Mediterranean (MED) diet, which emphasizes the intake of extra virgin olive oil, fruits, vegetables, whole grains, legumes, fish, nuts, and seeds. Specifically, a Mediterranean diet contains three to nine servings of vegetables, one half to two servings of fruit, 1 to 13 servings of cereals, and up to eight servings of olive oil daily (6). Adherence to a Mediterranean diet eating pattern can be quantified using a 14-point Mediterranean Diet Adherence Score (MEDAS) (7). A MED diet has been shown to reduce inflammation by lowering C-reactive protein and interleukin (IL)-6 levels and is associated with reduced obesity, cardiovascular disease, and cancer risk (8–11). Adherence to a MED diet has been found to alter the gut microbiome, which is the collection of bacteria, fungi, viruses, and other microorganisms living within the large intestine (12–17). Mechanistically, the dietary fiber and unsaturated fat in the MED diet are fermented by gut microbes, resulting in the production of anti-inflammatory metabolites (18). However, it remains to be seen whether a MED diet can be strategically used to manipulate the gut microbiota to promote health by reducing inflammation in MPN.

We performed a randomized clinical trial to investigate whether registered dietician counseling of individuals with MPN can alter their eating patterns toward a Mediterranean style. Subjects were randomly assigned to (i) MED diet counseling supplemented with complementary extra virgin olive oil (MED cohort) or (ii) diet counseling following the standard US Guidelines for Americans (USDA cohort) supplemented with grocery certificates. The study length was 15 weeks, consisting of a 2-week pre-intervention observation, 10 weeks of active dietary counseling, and 3 weeks of post-counseling follow-up. A description of the education-based MED intervention, patient satisfaction, diet adherence, symptom burden, and cytokine concentrations is available in a companion manuscript (19). As a key exploratory endpoint, we investigated whether a MED diet could produce a microbiome-mediated reduction in inflammation. Blood and stool samples were collected to measure cytokine levels and assess gut microbiome composition, respectively. Survey data were collected to assess the feasibility of a MED diet intervention among MPN patients and symptom burden was tracked using the MPN Symptom Assessment Form (MPN-SAF). In this manuscript, we detail the association of the gut microbiota with diet, MPN subtype, and cytokine concentrations.

## RESULTS

### Cohort description and study synopsis

**Fig 1:**
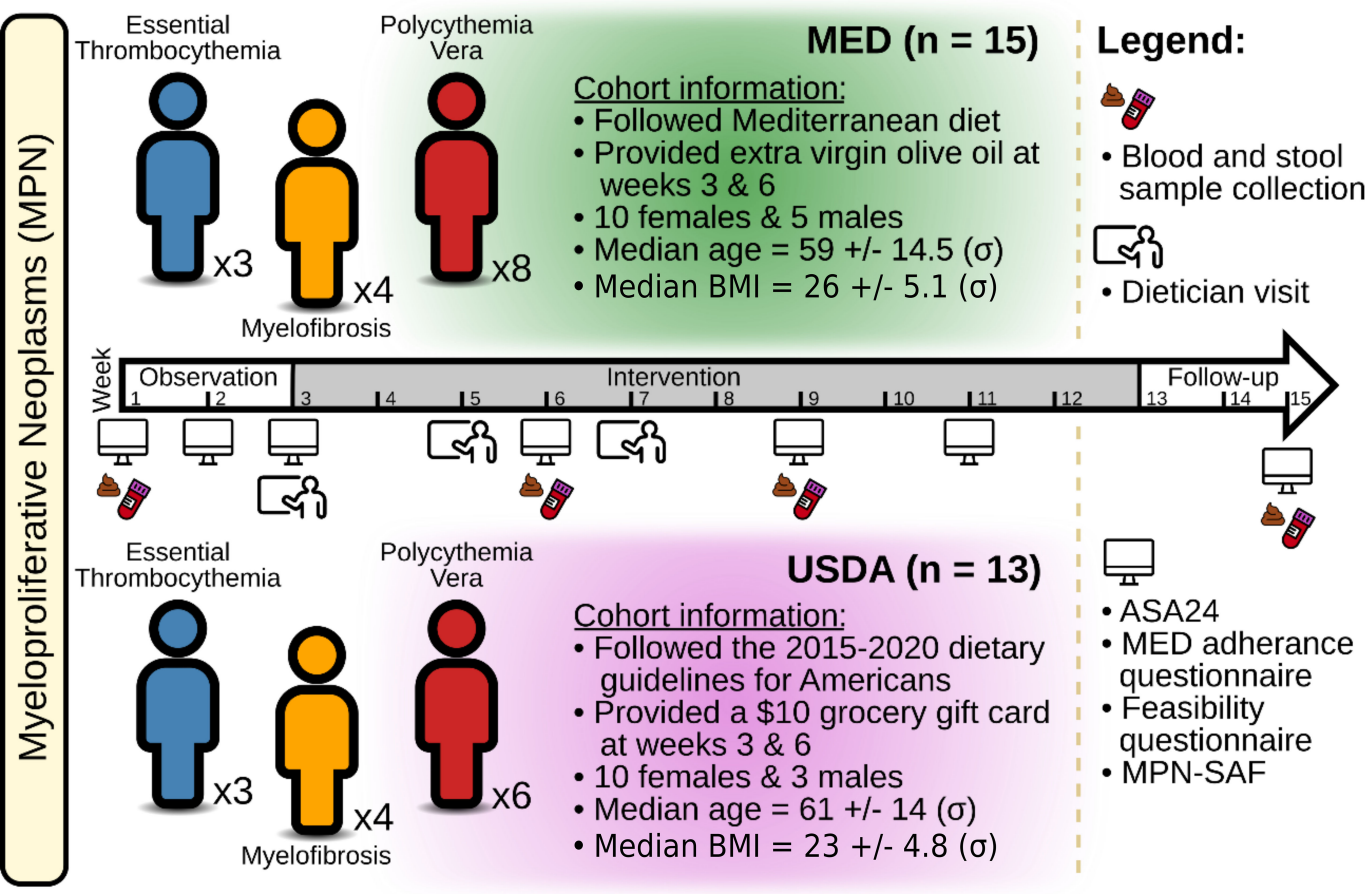
Study design. A total of 28 individuals with MPNs were enrolled in the study. Participants were randomly assigned to dietary counseling following either a MED diet (*n* = 15) or a conventional American diet (USDA, *n* = 13). The study was 15 weeks long and had a 2-week observation period, a 10-week intervention period, and a 3-week follow-up period. Blood and stool samples were collected at weeks 1, 6, 9, and 15. At weeks 3, 5, and 7, participants met with a dietician and were informed about the core components of each diet and how to follow it. On weeks 1, 2, 3, 6, 9, 11, and 15, subjects were asked to fill out 24-hour dietary recalls (ASA24), MED adherence and feasibility questionnaires, and an MPN-SAF.

In all, 28 subjects with MPN were recruited for this study (Fig. 1). The MED cohort had 15 individuals, while the USDA cohort had 13 individuals. Within the MED cohort, three subjects had ET, four had MF, and eight had PV. Within the USDA cohort, three subjects had ET, four had MF, and six had PV. The median age for the MED cohort was 59 ± 14.5 (σ) years, while the median age for the USDA cohort was 61 ± 14 (σ) years. The median BMI for the MED cohort was 26 ± 5.1 (σ) years, and the median BMI for the USDA cohort was 23 ± 4.8 (σ) years. Both groups had 10 females each, with 5 and 3 males in the MED and USDA cohorts, respectively. The study took place over 15 weeks and had an active intervention period from weeks 3 to 12. Baseline blood and stool samples were collected at week 1, followed by additional sampling during the active intervention at weeks 6 and 9. Follow-up samples were also taken after the intervention’s end at week 15. Throughout the study, six unannounced surveys and 24-hour food recalls (ASA24) were collected to measure diet compliance and symptom burden was assessed using the MPN-SAF, which grades the 10 most clinically relevant symptoms of MPN patients (20). Table 1 provides a detailed description of each subject’s characteristics.

**TABLE 1:** Subject characteristics

Subject	Diet	MPN	Treatment	Mutation	Age	Sex	BMI
2	USDA	PV	Ruxolitinib (Jakafi)	JAK2	71	M	30
3	USDA	MF	Observation only	MPL	63	F	23
5	USDA	MF	Hydroxyurea (Hydrea), interferon (Pegasys)	JAK2	63	F	22
7	USDA	PV	Interferon (Pegasys)	JAK2	44	F	25
9	USDA	ET	Observation only, aspirin	JAK2	57	M	26
10	USDA	PV	Observation only, phlebotomy	JAK2	21	F	24
12	USDA	PV	Hydroxyurea (Hydrea)	JAK2	77	M	36
14	MED	PV	Aspirin, interferon (Pegasys)	JAK2	34	F	32
15	MED	PV	Hydroxyurea (Hydrea)	JAK2	68	F	29
16	MED	ET	Aspirin, hydroxyurea (Hydrea), interferon (Pegasys)	JAK2	70	F	39
17	MED	PV	Eliquis and phlebotomy	JAK2	58	F	36
18	MED	PV	Hydroxyurea (Hydrea), prednisone	JAK2	66	M	24
19	USDA	ET	Aspirin	JAK2	61	F	32
20	MED	ET	Aspirin, hydroxyurea (Hydrea)	CALR	71	M	26
21	MED	MF	Aspirin, hydroxyurea (Hydrea)	CALR	25	F	22
22	MED	MF	Observation only, Chinese herbs	JAK2	71	F	25
23	MED	PV	Anagrelide (Agrylin), ruxolitinib (Jakafi)	JAK2	54	F	25
24	MED	MF	Ruxolitinib (Jakafi)	JAK2	67	F	21
25	MED	PV	Observation only, aspirin, other	JAK2	59	F	24
26	MED	MF	Observation only, Aspirin	JAK2	53	M	28
28	MED	PV	Aspirin, interferon (Pegasys)	JAK2	40	M	26
29	MED	ET	Aspirin, hydroxyurea (Hydrea)	JAK2	70	F	22
30	MED	PV	Observation only, aspirin	JAK2	50	M	27
31	USDA	ET	Aspirin, hydroxyurea (Hydrea)	JAK2	66	F	22
32	USDA	PV	Aspirin, hydroxyurea (Hydrea), other	JAK2	67	F	21
33	USDA	PV	Aspirin, hydroxyurea (Hydrea)	JAK2	57	F	22
34	USDA	MF	Other	JAK2	51	F	22
35	USDA	MF	Observation only	JAK2	58	F	20

### Impact of dietary intervention on consumed calories, body weight, macronutrients, and fiber intake

**Fig 2:**
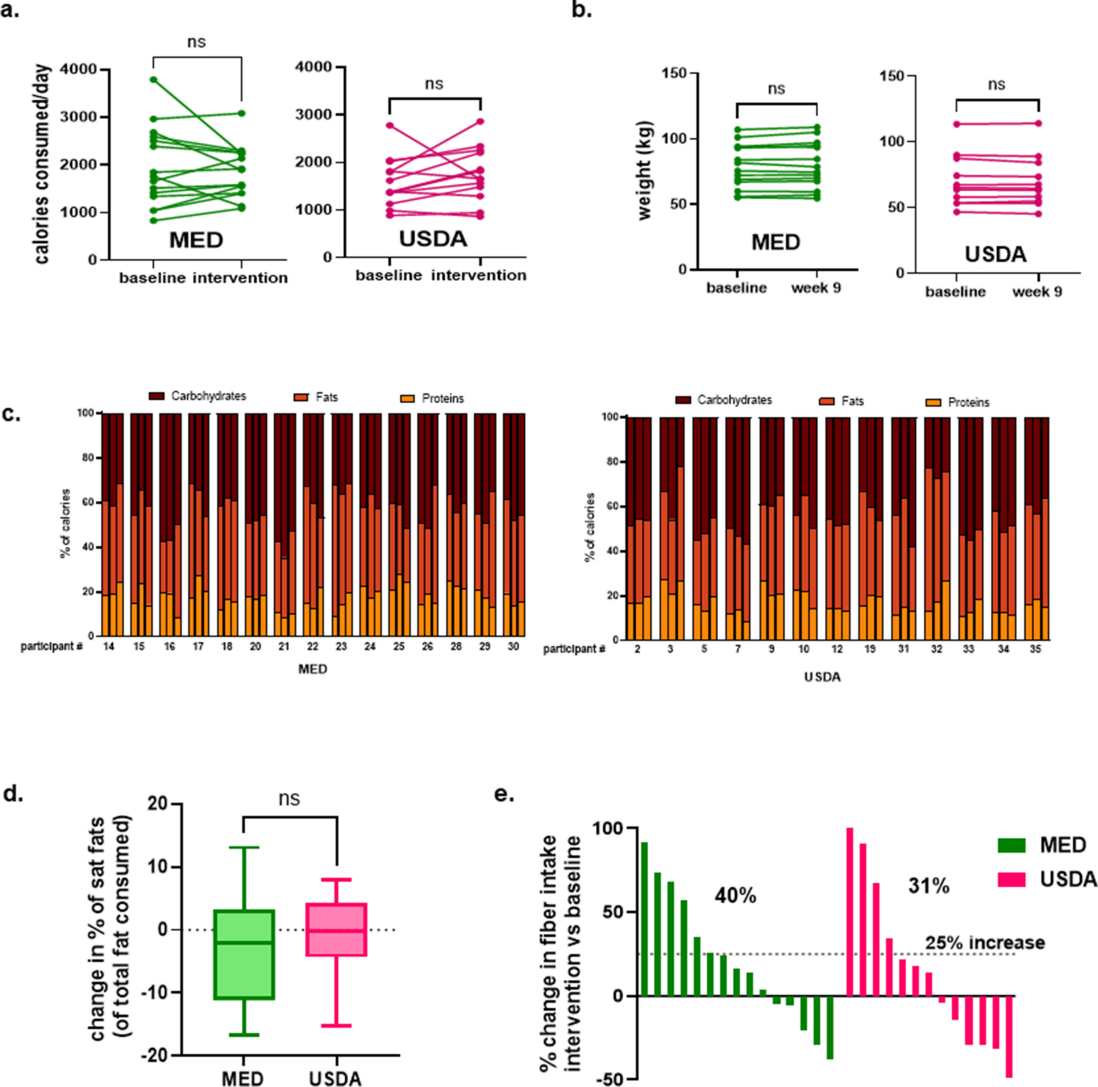
Impact of dietary intervention on consumed calories, body weight, macronutrients, and fiber intake. (**a**) Average calories consumed in each group at baseline (average of weeks 1 and 2) and during active intervention (average of weeks 6, 9, and 11). (**b**) Change in weight at week 9 compared to enrollment. (**c**) Visualization of the percentage of calories from carbohydrates, proteins, and fat each participant consumed at baseline (left bar) during active intervention (center bar), and follow-up period (right bar). (**d**) Change in percentage of calories from saturated fats during intervention (average of weeks 6, 9, and 11) vs baseline (average of weeks 1 and 2). (**e**) Waterfall plot of change in fiber intake during the active intervention period vs baseline. Each bar represents a participant. Significance testing was performed using a *t*-test.

Participants completed 24-hour diet recalls at weeks 1, 2, 3, 6, 9, 11, and 15. Data from weeks 1 and 2 were used to calculate average baseline intake and data from weeks 6, 9, and 11 were used to calculate average intake during the active intervention. Neither group had a significant change in total calorie intake during the active intervention period (Fig. 2a). A significant change in body weight was also not observed at week 9 when compared to baseline (Fig. 2b). A visual representation of the percentage of calories from carbohydrates, protein, and fat is shown in Fig. 2c. We investigated how each diet intervention impacted the percentage of calories from saturated fat. There was a mean change in the percentage of calories from saturated fat of −1.19% and −2.25% in the USDA and MED groups, respectively, which was not statistically significant (Fig. 2d). We also measured the change in fiber intake during the active intervention period. Thirty-one percent of the USDA group increased fiber intake by at least 25% and 40% of the MED group increased fiber intake by at least 25% (Fig. 2e).

### Gut microbiome diversity and composition are stable during the Mediterranean diet intervention

**Fig 3:**
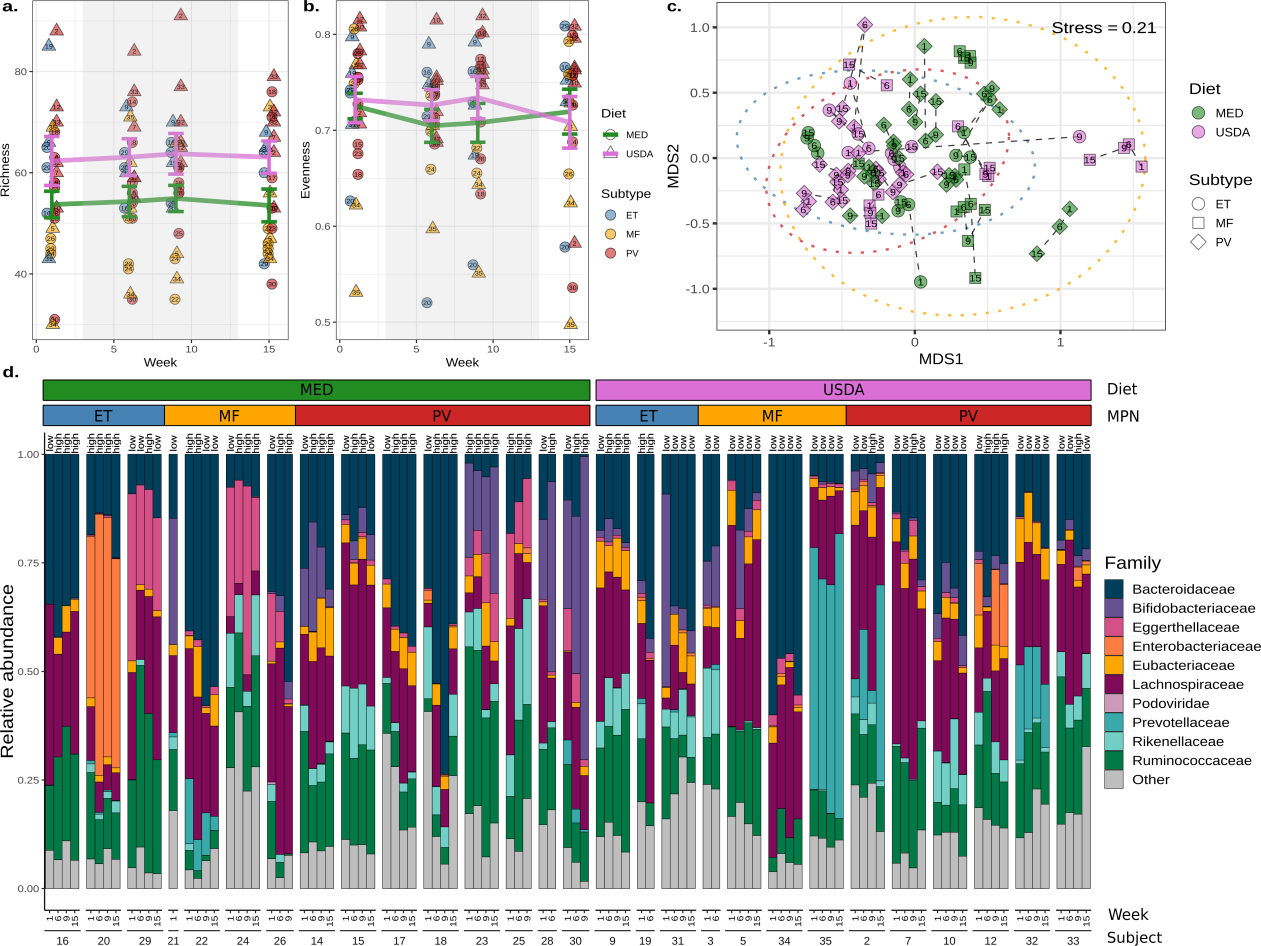
Gut microbiome diversity and composition are stable during the Mediterranean diet intervention. (**a**) Microbial richness and (b) evenness estimates of fecal samples collected at weeks 1, 6, 9, and 15. The shaded background indicates the active dietary intervention period for both diet groups. The mean richness or evenness for each group is represented with a colored line, with the error bars reflecting the standard error. Each point is labeled centrally with the individual of origin. (**c**) Non-metric multidimensional scaling of Bray-Curtis dissimilarities produced from compositional microbiome data. Points are colored by diet and shaped by the MPN subtype. A 95% confidence interval was drawn around each MPN subtype (blue = ET, yellow = MF, and red = PV). Dashed lines connect samples taken from the same individual, and the week of collection is labeled centrally within each point. (**d**) A taxa bar plot of the top 10 most abundant microbial families across individuals, time, diet, and MPN subtypes. Each sample is labeled “high” or “low” and refers to MED adherence for the week.

We began our investigation by examining how the MED diet impacts gut microbiome diversity. Analysis of species richness estimates using a linear mixed-effects model (LME) demonstrated that USDA and MED groups did not significantly differ over time after accounting for pre-intervention differences (LME *P*-value = 0.48, Fig. 3a). Analyses of species evenness estimates also showed no differences between diet groups (LME *P*-value = 0.65, Fig. 3b). Sub-setting species richness and evenness comparisons to include only samples from participants highly adherent to a Mediterranean style eating pattern and those least adherent to a Mediterranean style eating pattern during the intervention also did not reveal significant differences (LME richness *P*-value = 0.48, LME evenness *P*-value = 0.73).

Next, we examined the microbial composition, or beta-diversity, of the fecal samples. Species composition analysis using non-metric multidimensional scaling (NMDS) and permutational multivariate analysis of variance (PERMANOVA) showed that there were significant differences associated with MED and USDA groups pre-intervention (Fig. 3c; PERMANOVA *R^2^* = 0.057, *P*-value = 0.046, Table S1). Therefore, we stratified our PERMANOVA analysis to investigate whether gut microbiome composition changed over time within each individual. This produced non-significant results, showing that microbiome composition was stable throughout the study (Fig. 3d; PERMANOVA *R^2^* = 0.007, *P*-value = 0.76, Table S2). Next, PERMANOVA was performed on each MPN subtype to examine whether a specific subtype responded to the diet intervention more than others. No changes were detected in ET (PERMANOVA *R^2^* = 0.046, *P*-value = 0.63), MF (PERMANOVA *R^2^* = 0.026, *P*-value = 0.60), or PV (PERMANOVA *R^2^* = 0.016, *P*-value = 0.77) subtypes over time (Table S3). Consequently, no microbial species were significantly different between MED and USDA groups after adjusting for pre-existing compositional differences.

Characterization of the functional metagenome demonstrated no significant differences between diets as measured by microbial gene richness (LME *P*-value = 0.65, Fig. S1A) and gene evenness (LME *P*-value = 0.19, Fig. S1B) after accounting for pre-intervention differences. PERMANOVA analysis indicated that there were no significant changes over time within each individual (PERMANOVA *R^2^* = 0.009, *P*-value = 0.29, Table S4). No differentially abundant genes were found between MED and USDA groups.

### Individuals with myelofibrosis have reduced microbial diversity and altered composition

Previous research has demonstrated significant differences in the microbiomes associated with healthy individuals and those with MPN (21). Therefore, we characterized the microbiome between PV, ET, and MF subtypes further. Using species richness estimates, we observed a significant reduction in the number of unique microbes when comparing individuals with MF to PV (linear mixed-effects *P*-value = 0.028, Fig. S2A), and a non-significant reduction when comparing MF to ET (linear mixed-effects *P*-value = 0.056, Fig. 3a). Species abundance distribution, or evenness, was also reduced in MF, but was not significant compared to PV (LME *P*-value = 0.12) and ET subtypes (LME *P*-value = 0.47, Fig. 3b).

**Fig 4:**
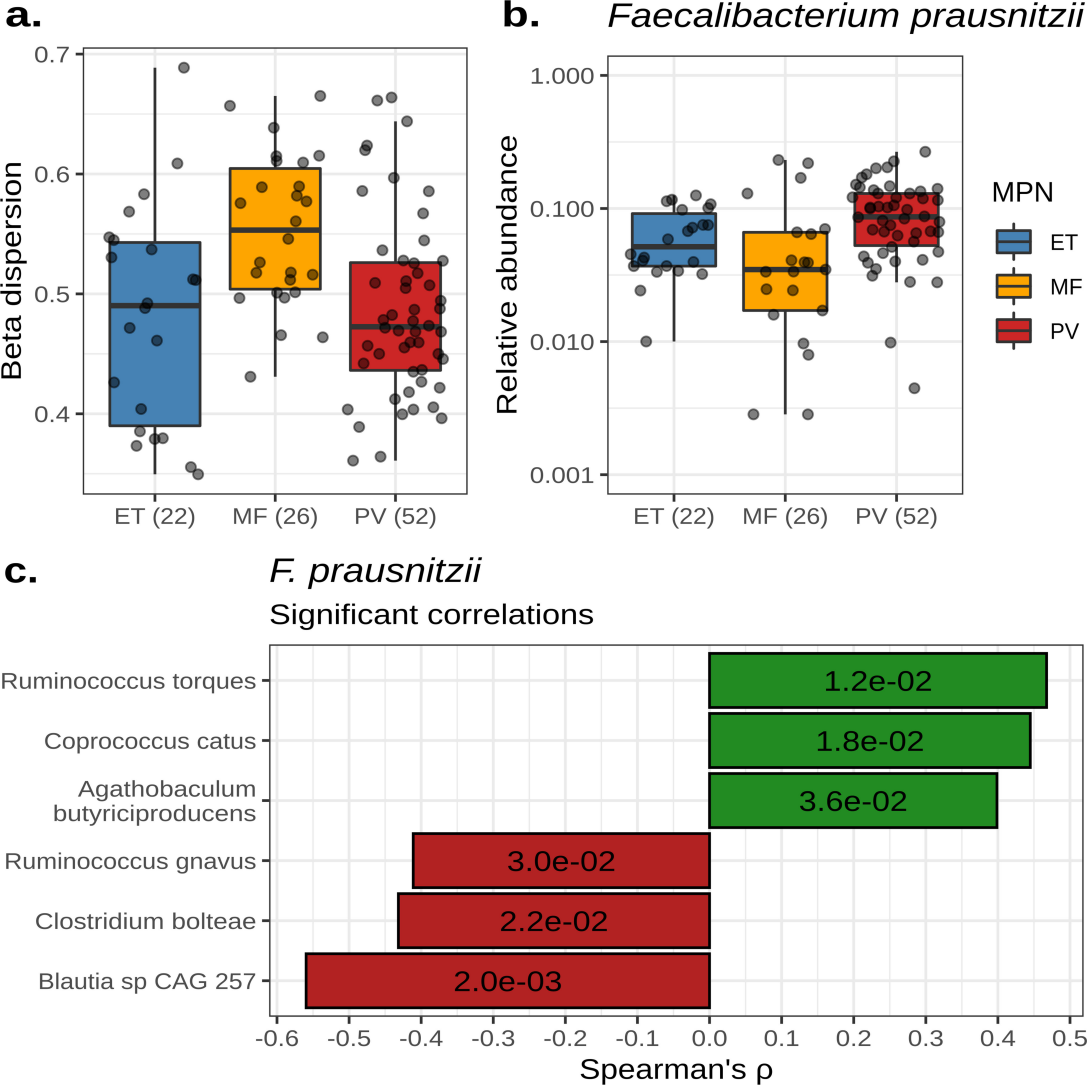
Individuals with myelofibrosis have reduced microbial diversity and altered composition. (**a**) A box plot showing the beta-dispersion of each MPN subtype calculated from taxonomic Bray-Curtis dissimilarities. (**b**) The relative abundance of *Faecalibacterium prausnitzii* across MPN subtypes. For (A) and (B), the number of samples per subtype is labeled parenthetically and the center line within each box defines the median. Boxes define the upper and lower quartiles and whiskers define 1.5× the interquartile range. (**c**) A bar plot showing the Spearman correlation coefficients of microbes significantly correlated with *F. prausnitzii* abundance. *P*-values for each correlation are labeled within each bar.

With respect to beta-diversity, samples from MF were more dissimilar from each other, resulting in a trend toward increased beta-dispersion when compared to ET (LME *P*-value = 0.089) and PV (LME *P*-value = 0.056, Fig. S2C; Fig. 4a). Conversely, PV and ET samples tended to cluster together (LME *P*-value = 0.895, Fig. S2C; Fig. 4a). PERMANOVA demonstrated that the individual of origin significantly explained about 51% of the variance observed in the microbiome, while the MPN subtype significantly explained approximately 6.0% of variance (PERMANOVA *P*-value = 0.001 for both, Table S5). For comparison, age, sex, and BMI explained 3.3%, 3.3%, and 2.4% of the variance associated with microbiome composition, respectively (PERMANOVA *P*-values = 0.001, Table S5). The analysis of microbial composition between MPN subtypes showed a reduction in the abundance of *Faecalibacterium prausnitzii* in MF subjects when compared to those with PVor ET (ANCOM2 *P* < 0.05, Fig. 4b). Microbes most correlated with *F. prausnitzii* abundance included *Ruminococcus torques, Coprococcus catus, Agathobaculum butyriciproducens, Ruminococcus gnavus, Clostridium bolteae,* and *Blautia sp*. CAG-257 (Fig. 4c).

Within the functional metagenome data, we detected a significant reduction in the number of unique microbial genes within MF subjects when compared to PV (LME *P*-value = 0.016, Fig. S3A), but not ET (linear mixed-effects *P*-value = 0.244, Fig. S3A). There was no significant difference in the gene evenness among MPN subtypes (Fig. S3B). NMDS ordination demonstrated that the functional metagenome compositions of MF samples tended to be more disparate from each other when compared to PV (LME *P*-value = 0.34) and ET (LME *P*-value = 0.19, Fig. S3C and D). In addition, the MPN subtype significantly explained about 6.7% of the variance observed in functional metagenome composition (PERMANOVA *P*-value = 0.001, Table S6), while the individual of origin was associated with about 51% of the variance (PERMANOVA *P*-value = 0.001, Table S6). Differential abundance analysis produced no significantly different genes between MPN subtypes after FDR correction.

### Cytokine levels are correlated with microbiome diversity and composition

**Fig 5:**
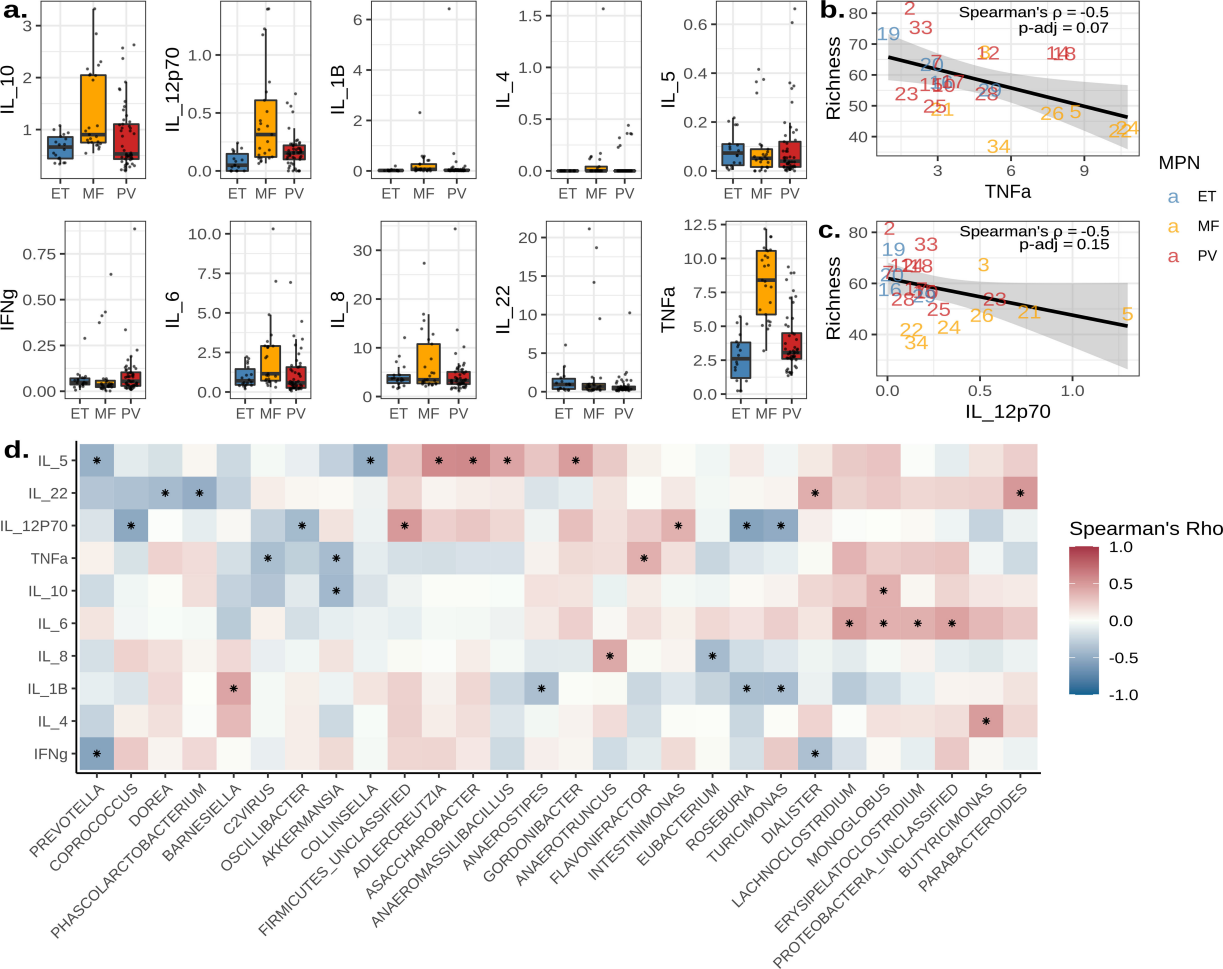
Cytokine levels are correlated with microbiome diversity and composition. (**a**) Box plots displaying the concentration of cytokines measured in pg/mL across MPN subtypes. The center line within each box defines the median, boxes define the upper and lower quartiles, and whiskers define 1.5× the interquartile range. (**b and c**) Scatter plots of TNFα (**b**) and IL-12p70 (**c**) concentrations in pg/mL correlated with species richness estimates. Points are labeled by the individual of origin and colored by MPN subtypes. A line represents the mean, and the shaded area delineates the 95% confidence interval. (**d**) A heat map of microbial genera whose abundances significantly correlated with cytokine concentrations. Asterisks denote significant correlations (*P* < 0.05).

After subsequent analysis of MPN subtypes and their gut microbiomes, we next asked if the diversity or composition of the microbiome was associated with the concentrations of 10 plasma cytokines. Comparison of cytokine concentrations between MPN subtypes revealed a significant increase in TNFα and IL-12p70 in subjects with MF when compared to ET (Tukey’s test; TNFα *P*-adj <0.001 and IL-12p70 *P*-adj = 0.016) and PV (Tukey’s test; TNFα *P*-adj = 0.002 and IL-12p70 *P*-adj = 0.022, Fig. 5a). IL-6, IL-8, and IL-10 concentrations were elevated in subjects with MF but were not statistically significant (Fig. 5a). Microbial richness was most negatively correlated with TNFα (Spearman’s ρ = −0.50, *P*-adj = 0.07, Fig. 5b) and IL-12p70 (Spearman’s ρ = −0.45, *P*-adj = 0.15, Fig. 5c).

Next, we compared cytokine concentrations with microbial abundances at the genus level, resulting in 34 significant correlations (Fig. 5d). Notable correlations included associations with TNFα vs *Flavonfractor* (Spearman’s ρ = 0.39, *P* = 0.038), IL-12p70 vs *Roseburia* (Spearman’s ρ = −0.55, *P* = 0.002), and IL-8 vs *Eubacterium* (Spearman’s ρ = −0.41, *P* = 0.032, Fig. S4). Similarly, we compared cytokine concentrations with functional pathway abundances, producing 162 significant correlations (Fig. S5). Notable correlations included TNFα vs 4-deoxy-L-threo-hex-4-enopyranuronate degradation (Spearman’s ρ = −0.42, *P* = 0.038), TNFα vs β-(1,4)-mannan degradation (Spearman’s ρ = −0.48, *P* = 0.019), and IL-12p70 vs GDP-mannose biosynthesis (Spearman’s ρ = −0.59, *P* = 0.003, Fig. S6).

## DISCUSSION

Our goal with this manuscript was to (i) assess whether a MED diet altered the gut microbiome of subjects with MPN and (ii) investigate the association between diversity and composition of the gut microbiome and levels of plasma cytokines. In a separate manuscript, we describe the feasibility of a MED diet intervention in the MPN subject population, changes in macronutrients associated with the dietary intervention, and the interaction between diet adherence, symptom burden, and cytokine concentrations (19). Here, we report that microbial diversity or composition did not significantly change during a MED diet intervention over a 10-week active dietary intervention period. Instead, we found the MPN subtype played a greater role in determining microbiome diversity and composition. Individuals with ET and PV had more similar microbial compositions, while those with MF were more disparate. Furthermore, a reduction in microbial diversity correlated with elevated TNFα and IL-12p70 concentrations in subjects with MF. These differences in cytokine concentrations were associated with the abundances of 34 microbial genera and 162 metabolic pathways, further establishing a role for the gut microbiome in inflammation and MPN.

With respect to diet-mediated changes in the microbiome, there are multiple potential explanations as to why the microbiomes of individuals remained stable throughout the dietary intervention. The first is intervention duration. Long-term adherence to a Mediterranean diet has been demonstrated to reduce the incidence of cardiovascular disease, Alzheimer’s disease, colorectal cancer, diabetes, and obesity (10, 11). Studies performing MED diet interventions have ranged from 6 weeks to 7 years (14–17, 22–30). Of studies examining gut microbiome composition, the 6-week MED diet intervention performed by Marlow et al. yielded no significant differences in gut microbiome composition or CRP levels in subjects with Crohn’s disease (30). Comparatively, Haro et al. conducted a MED diet intervention in a cohort of 20 obese men and observed differences in microbiota composition after 1 year (17). Similarly, Nagpal et al. conducted a MED diet intervention in a non-human primate model over the course of 2.5 years, and a significant difference in the microbiome was observed between macaques who consumed a Western diet vs a MED diet (16). Because this was a feasibility study, and the primary objective was to assess whether patients with MPN could change their diet with dietician counseling, a relatively short duration was chosen. Our data and others suggest that longer MED dietary intervention periods are needed to detect changes in gut microbiome composition.

Another consideration in the successful manipulation of gut microbiomes with diet is the presence of specific microbial taxa, functions, or enterotypes. Stratification of microbiomes into enterotypes has previously revealed enterotype-specific predictors of dietary intervention response (31). Klimenko et al. found that the strongest predictor of whether an individual would respond to a dietary intervention was the average number of genes per microbe (31). A negative correlation between the average number of genes per microbe and alpha diversity was found, suggesting that more diverse communities are formed by specialist microbes with fewer genes (31). The microbiomes associated with industrialized countries, like the United States, often have reduced diversity and a higher abundance of *Bacteroides* when compared to non-industrialized countries (32). Many *Bacteroides* are generalists, meaning they contain more genes and wider metabolic potentials than specialist taxa (33). The predominance of generalist taxa has been known to contribute to microbiome stability (34). Therefore, it is plausible that the microbiomes of industrialized individuals have evolved to resist perturbations, such as those caused by antibiotic usage or short-term dietary changes. Our samples contained relatively high abundances of *Bacteroides*, so it is possible that the microbiomes of these individuals were resistant to short-term dietary changes as reflected by the non-significant changes in diversity, composition, and function over time.

A final factor that could have affected the stability of our microbiomes is the strength of dietary intervention. Due to differences in agriculture and food processing, a MED diet in the United States is different from a MED diet in the Mediterranean region. This can affect the number of antibiotic and prebiotic compounds found in each diet. One prebiotic component of the MED diet that can influence gut microbiome composition is extra virgin olive oil (EVOO). EVOO is rich in polyphenols and oleic acid, which have been demonstrated to have anti-oxidative and anti-inflammatory properties (35, 36). Over 90% of polyphenols are digested and metabolized in the colon by the gut microbiota (37). Dietary supplementation of EVOO in humans has been shown to promote the growth of beneficial microbes like *Bifidobacterium* and lactic acid-producing bacteria (35, 38). In rodent models, consumption of EVOO results in an increased abundance of *Bifidobacterium, Lactobacillus,* and *Clostridium* (39, 40). Our future dietary interventions in MPN patients will include an educational module on the benefits of EVOO, which we anticipate will lead to increased EVOO consumption and a change in gut microbiome composition.

A MED diet is also typically higher in dietary fiber when compared to a typical USDA diet. Dietary fiber is fermented by the gut microbiota to produce short-chain fatty acids, such as acetate, propionate, and butyrate. Butyrate is critical for gut health, as it is the primary source of energy for colonocytes and reduces inflammation by stimulating the production of T-regulatory cells and IL-10-producing cells (41). In this study, a minority of participants increased their fiber intake during the intervention period. As such, we did not find that the abundances of butyrate-producing bacteria differed between diets. Instead, we saw a reduction in the butyrate-producing microbe, *F. prausnitzii,* in subjects with MF. We also noted significant positive correlations between *F. prausnitzii, Agathobaculum butyriciproducens,* and *Coprococcus catus* abundances. *A. butyriciproducens* is a butyrate-producing microbe, while *C. catus* produces both butyrate and propionate (42). We also observed broader, community-wide differences between subjects with ET, PV, and MF. Notably, the microbiome composition of MF subjects was more dissimilar to each other when compared to ET and PV. Our previous work comparing the gut microbiome composition of healthy and MPN subjects similarly showed that individuals with MF had increased beta-dispersion when compared to ET and PV (21). These results describe a phenomenon known as the “Anna Karenina principle” for animal microbiomes, which states that stressors affect microbiomes in unpredictable ways, leading to increased community beta-dispersion (43, 44).

One likely stressor resulting in higher MF beta-dispersion is the increased concentration of pro-inflammatory cytokines. Inflammation has been known to negatively affect the gut microbiome. Supporting this notion, we found that TNFα and IL-12p70 were significantly increased in MF subjects, which negatively correlated with species richness overall. We found IL-12p70 negatively correlated with the genus *Roseburia*, which are butyrate-producing microorganisms known to alleviate inflammation by promoting T-regulatory cell differentiation (45, 46). We also observed a significant negative correlation with *Eubacterium* and the pro-inflammatory cytokine, IL-8. *Eubacterium* also produces butyrate and has been shown to lessen inflammation by promoting IL-10 production (45, 47). TNFα plasma concentrations were found to be negatively correlated with the abundance of the 4-deoxy-L-threo-hex-4-enopyranuronate degradation pathway, which aids in the degradation of uronic acids, which includes pectin fiber. Another fiber whose degradation pathway was negatively correlated with TNFα was β-(1,4)-mannan.

Taken together, it is possible that the increased inflammation observed in individuals with MPN, particularly MF, is exacerbated by the lack of dietary fiber and short-chain fatty acid production. The MED diet has been previously shown to promote the growth of *F. prausnitzii* specifically; therefore, future experiments could attempt to restore microbial short-chain fatty acid production to reduce inflammation. When designing dietary interventions, however, special attention should be given to the intervention duration and the ability of existing gut microbes to use and respond to prebiotic compounds. This may ensure that the desired outcomes are achieved, allowing us to manipulate the gut microbiome to promote health and ameliorate disease.

## MATERIALS AND METHODS

### Recruitment of subjects

Patients were recruited between October 2018 and September 2019. Participants were included if they were over the age of 18 with a previous diagnosis of a Philadelphia chromosome-negative MPN (including PV, ET, and MF), had an ECOG score of 2 or less, a life expectancy of greater than 20 weeks, had internet access, an email address, and could read and understand English. Any type of previous or current therapy was also allowed. Participants were excluded if they were pregnant or planning on becoming pregnant, lost more than 10 pounds or 10% of their body weight in the last 6 months, or were allergic to nuts and olive oil. In all, 47 participants were screened. Five did not meet the inclusion criteria, and an additional 11 subjects were excluded due to incomplete survey data during the observation period. In all, 31 subjects were randomly assigned to a diet, but two withdrew participation and one failed to provide sufficient survey data. The final number of study participants was 28, with 15 belonging to the MED cohort and 13 belonging to the USDA cohort.

### Collection of dietary intervention feasibility, adherence, and symptom burden data

During the first week of the intervention period, each participant met individually with a dietician to learn about the central components of their assigned diet. There were follow-up dietician visits during weeks 5 and 7. Participants were emailed educational materials on their respective diets weekly during the 10-week active intervention period. Furthermore, participants in the MED cohort were given 750 mL of extra virgin olive oil, and those in the USDA cohort were given a $10 grocery gift card at weeks 3 and 6. Throughout the study, participants were required to fill out 4 unannounced surveys given during weeks 1, 2, 3, 6, 9, 11, and 15. The first survey measured dietary intervention feasibility and asked, “how easy is it for you to follow this diet, with 1 being very easy to follow and 10 being very difficult to follow?” The second survey measured MED diet adherence. For this, the established 14-item Mediterranean diet adherence score (MEDAS) was used (7). Adherence to a MED diet was defined as a “high” for the week if a score of >8/14 was obtained. Next, we asked subjects to complete 24-hour food recalls using the Automated Self-Administered 24-hour Dietary Assessment Tool (ASA24), providing detailed information about calories consumed, macronutrients, and dietary fiber. Lastly, symptom burden was assessed *via* the MPN-SAF, which grades the 10 most clinically relevant MPN symptoms (20). Surveys were administered through email.

### Blood collection and cytokine measurements

Peripheral blood was drawn on weeks 1, 3, 6, and 15 in tubes containing ethylenediaminetetraacetic acid. Plasma was obtained by centrifuging 3–4 mL of blood for 10 min at 2,500 rpm, aliquoted, and stored at −80°C. Frozen plasma was sent to Quanterix in Billerica, MA for analysis. A Human CorPlex 10 Cytokine Array kit #85–0329 (IL-12p70, IL-1B, IL-4, IL-5, IFNɣ, IL-6, IL-8, IL-22, TNFα, and IL-10) was used according to the manufacturer’s protocol and analyzed using a Quanterix SPX imager system on-site at Quanterix Headquarters in Billerica, MA.

### Fecal sample collection

To perform gut microbiome analysis, four stool samples were requested from each participant over the course of the 15-week trial. The samples were collected by the participants themselves using Zymo RNA/DNA shield fecal collection tubes (Cat. #R1101) during weeks 1, 6, 9, and 15. Samples were returned in person or by mail. In total, 103 samples were collected. Samples were stored at −80°C once returned.

### DNA extraction

Fecal samples stored in a DNA/RNA shield were thawed on ice, vortexed to homogenize, then DNA from 1,000 uL of fecal slurry was extracted using the ZymoBiomics DNA Miniprep Kit (Cat. #D4300) according to the manufacturer’s protocol. Bead lysis during the extraction was performed at 6.5 m/s for 5 min total (MPBio FastPrep-24).

### Shotgun library preparation and sequencing

Libraries for shotgun sequencing were prepared using the Illumina DNA prep kit (Cat. # 20018705), using an adapted low-volume protocol (48). In summary, we reduced the amount of DNA used per sample to a maximum of 5 uL or 50 ng (whichever was reached first). Tagmentation was performed according to the manufacturer’s protocol, but volumes were reduced to 1 uL of bead-linked transposome and tagmentation buffer each. Next, 1.25 uL of 1 uM i5 and i7 indices were added to each sample and annealed *via* polymerase chain reaction using 10 uL of KAPA HiFi HotStart ReadyMix (Cat. # 7958935001). Afterward, libraries were combined, size selected, and cleaned using 56 and 14.4 uL of sample purification beads according to the low-volume protocol. Positive and negative sequencing controls were included during the library preparation using the ZymoBIOMICS Microbial Community DNA Standard (Cat. #D6305) and purified water, respectively. The quality of libraries was assessed with the Quanti-iT PicoGreen dsDNA (Cat. #P7589) for quantity and Agilent Bioanalyzer High Sensitivity DNA Analysis (Cat. #5067–4626) for fragment size. Libraries were shipped overnight on dry ice to Novogene Corporation Inc. (Sacramento, CA) to be sequenced using Illumina’s Hiseq 4000. An average of 2,819,107 ± 670,543 (σ) paired-end reads per sample, 150 base pairs long, were obtained.

### OTU table generation

Raw data were first cleaned to remove sequencing adapters and artifacts using the BBMap v38.79 script “bbduk.sh” with the flag “ref = adapters,artifacts” (49). BBMap’s “demuxbyname.sh” was used to demultiplex sequences using the default parameters. Quality filtering of sequences was performed using PRINSEQ ++ v1.2 with the following parameters: -trim_left 5 -trim_right 5 - min_len 100 -trim_qual_right 28 - min_qual_mean 25 (50). Quality checking was done with FastQCv0.11.8 on default parameters. This resulted in a mean and standard deviation of 2,731,886 ± 648,042 paired-end reads, respectively. Human-derived reads were removed using BowTie2 v2.4.5 using the default parameters and hg38 as the reference genome, which produced an average of 2,498,159 ± 960,477 (σ) reads per sample (51). Taxonomic assignment of sequence data was performed using MetaPhlAn v3.0.14 with the default parameters and the CHOCOPhlAn v2019.01 database (52).

### Microbiome functional potential data generation

Individual gene annotations were produced by first cross-assembling reads into contiguous sequences using MEGAHIT v1.1.1 with a minimum length of 2,500 base pairs and the flag “--k-list 31,41,51,61,71,81,91,101,111” (53). Afterward, open reading frames were assigned with Prodigal v2.6.3 and then annotated with eggNOG mapper v2.0 using the eggNOG v5.0 database (54, 55). Next, BowTie2 v2.4.5 was used to align samples to the annotated genes to obtain a table of per sample counts for each gene. Lastly, per-sample gene counts were normalized to reads per kilobase per genome equivalent using MicrobeCensus v1.1.1 on default parameters (56). For the functional annotation of metabolic pathways, we ran our quality-filtered, unassembled reads through HUMAnN v3.0.1 using the default parameters and the UniRef90 v201901b database (52). The “humann_renorm_table” and “humann_join_tables” scripts were used to create a pathway abundance table of normalized counts in copies per million.

### Data analysis

Data analysis of OTUs, genes, and pathways was performed in R v4.2.1. The first step was removing microbes or genes that contaminated our sequencing controls from all samples. The Vegan v2.6–2 package was used to calculate the following metrics: richness with the “specnumber” function, evenness with the formula “diversity(x, index = “Shannon”)/log_10_(specnumber(x)),” Bray-Curtis beta-diversity with the “vegdist” function, PERMANOVA with the “adonis2” function, NMDS with the “metaMDS” function, and beta-dispersion with the “betadisper” function. Please see Tables S1 to 6 for PERMANOVA formulas and parameters. Significance testing of richness, evenness, and beta-dispersion was performed using linear mixed-effect models with the nlme v3.1–159 package. Significance testing of cytokine concentrations was done using an ANOVA and Tukey’s post hoc test with the “aov” and “TukeyHSD” functions. Spearman correlations were obtained using the “cor.test” and “rcorr” functions. Differential abundance of OTUs was determined with ANCOM v2.1 with the parameters: rand_formula = “~ 1 | Subject,” p_adj_method = “none,” alpha = 0.05. For gene and pathway abundances, we averaged abundances within each subject to eliminate repeated measurements and performed a Kruskal-Wallis test. When appropriate, multiple comparisons were corrected using the “p.adjust(x, method = “fdr”)” function. All code, scripts, and parameters for data processing and analysis can be found at https://github.com/Javelarb/MPN_diet_intervention.

## Data Availability

Sequencing data are available on the Sequence Read Archive under the BioProject ID PRJNA918651. The taxonomic table with corresponding metadata used to generate the source data can be found in Supplementary_file_2.csv. Larger files used in our functional metagenomic analysis are available on the Dryad Digital Repository (doi:10.7280/D1DT3F). Additional data and materials are available upon reasonable request. All code for data processing and analysis are available on GitHub.

## SUPPLEMENTAL MATERIAL

File S1 mbio.02308-23-s0001.docx Figures S1-S6 and Tables S1-S6.

File S2 mbio.02308-23-s0002.csv Taxonomic table and metadata used for the analysis.

Supplemental legend mbio.02308-23-s0003.docx Legend for File S2.

ASM does not own the copyrights to Supplemental Material that may be linked to, or accessed through, an article. The authors have granted ASM a non-exclusive, world-wide license to publish the Supplemental Material files. Please contact the corresponding author directly for reuse.
