# Supplementary material for: Characterizing the microbiome of patients with myeloproliferative neoplasms during a Mediterranean diet intervention
Source: mBio. 2023 Oct 25;14(6):e02308-23. doi: 10.1128/mbio.02308-23 (PMC10746218; doi:10.1128/mbio.02308-23)
Supplement: File S1 — Figures S1-S6 and Tables S1-S6. [file mbio.02308-23-s0001.docx]

**Supplementary Figures:**


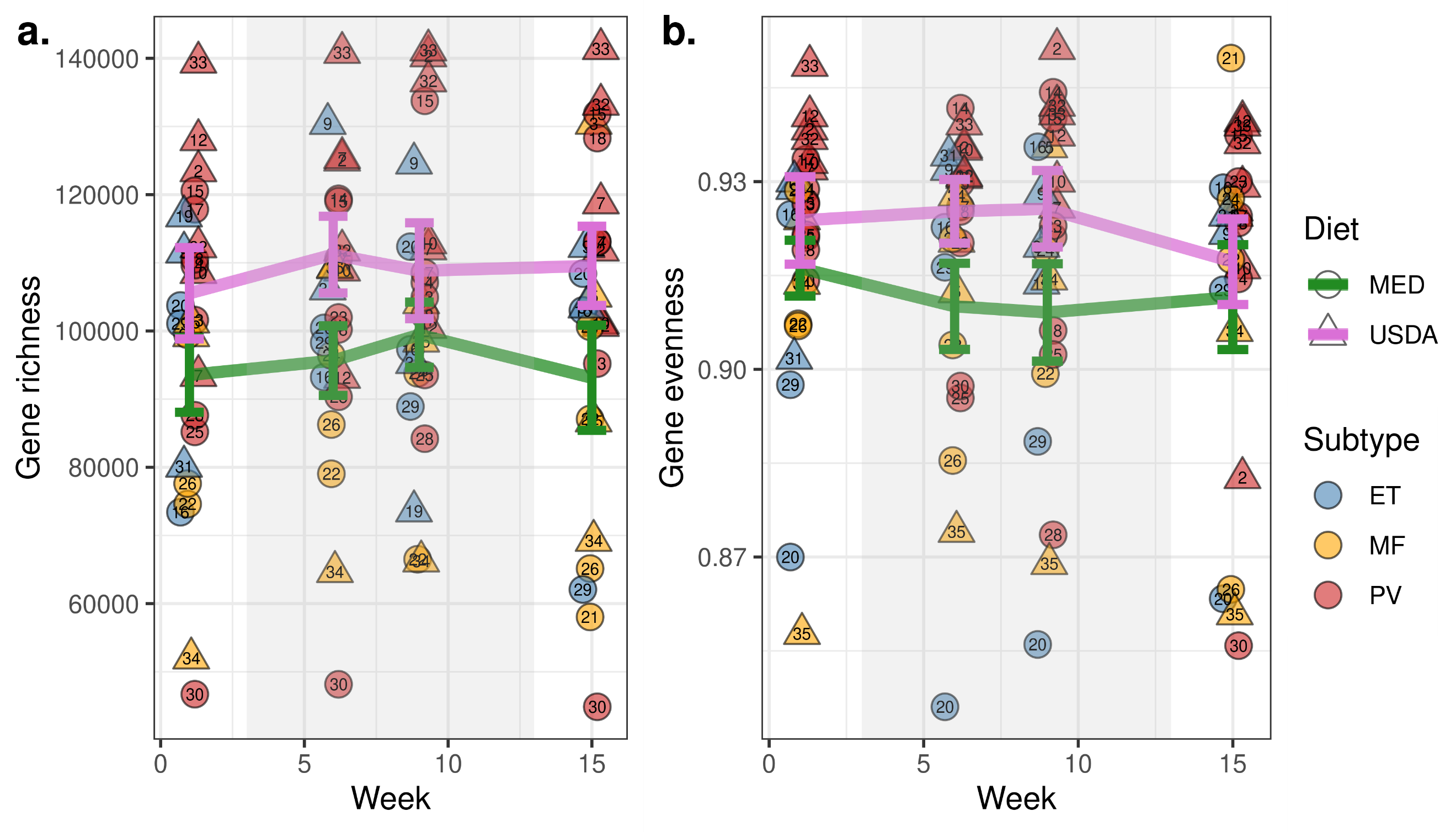


***Supplementary Figure 1:*** **A)** Gene richness and **B)** evenness estimates of fecal samples collected at weeks 1, 6, 9, and 15. The shaded background indicates the active dietary intervention period for both diet groups. The mean richness or evenness for each group is represented with a colored line, with the error bars reflecting the standard error. Each point is labeled centrally with the individual of origin.


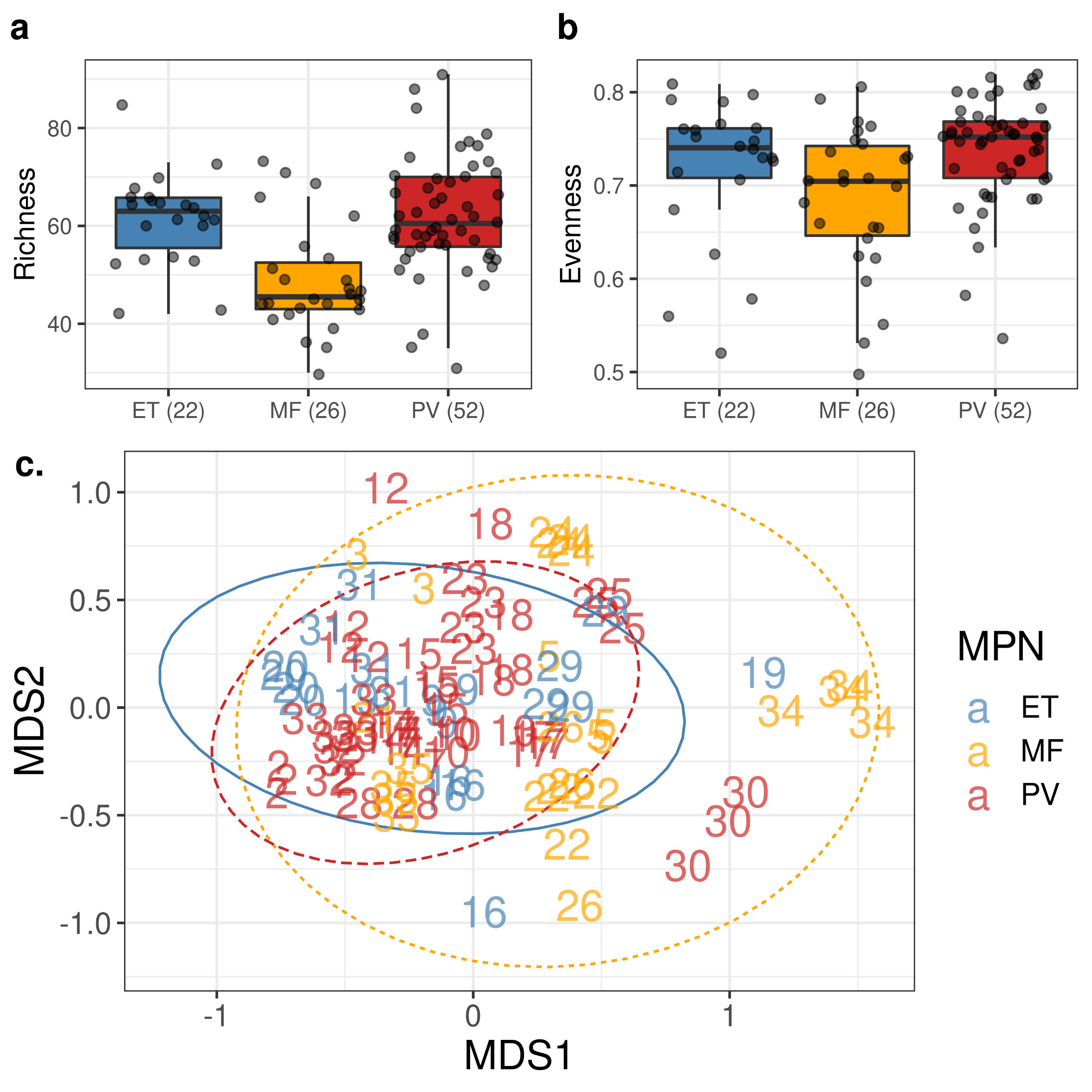


***Supplementary Figure 2:* A** and **B)** Box plots showing microbial richness and evenness estimates across MPN subtypes. The number of samples per subtype is labeled parenthetically and the center line within each box defines the median. Boxes define the upper and lower quartiles and whiskers define 1.5x the interquartile range. **C)** Non-metric multidimensional scaling of Bray-Curtis dissimilarities produced from compositional microbiome data. Points are labeled by individual and colored by MPN subtype. A 95% confidence interval was drawn around each MPN subtype.


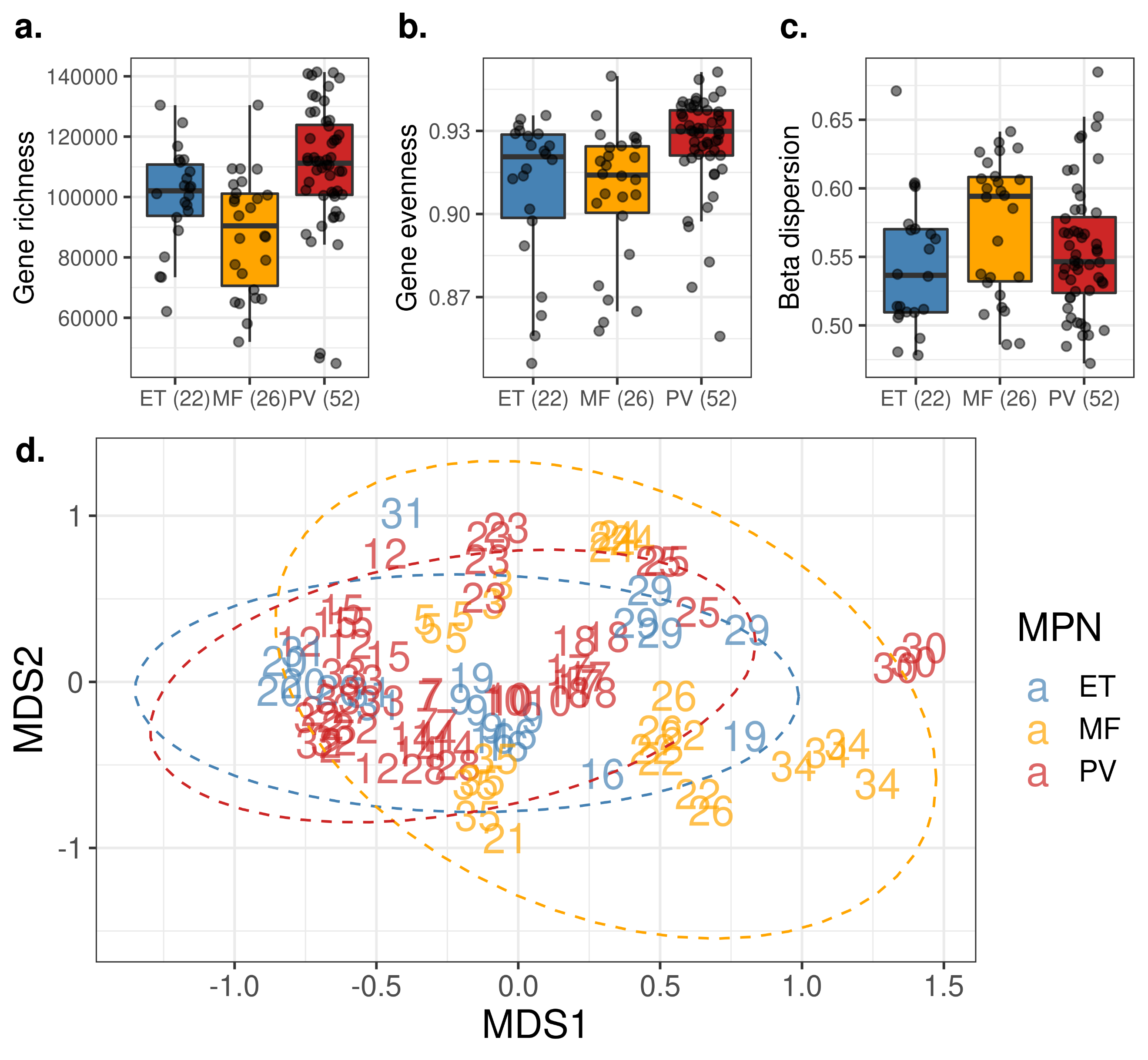


***Supplementary Figure 3:* A** and **B)** Richness and evenness box plots of microbial gene abundances. **C)** A box plot showing the beta dispersion of each MPN subtype calculated from gene Bray-Curtis dissimilarities. For A-C the number of samples per MPN subtype is labeled parenthetically and the center line within each box defines the median. Boxes define the upper and lower quartiles and whiskers define 1.5x the interquartile range. **D)** Non-metric multidimensional scaling of Bray-Curtis dissimilarities produced from compositional gene data. Points are labeled by individual and colored by MPN subtype. A 95% confidence interval was drawn around each MPN subtype.


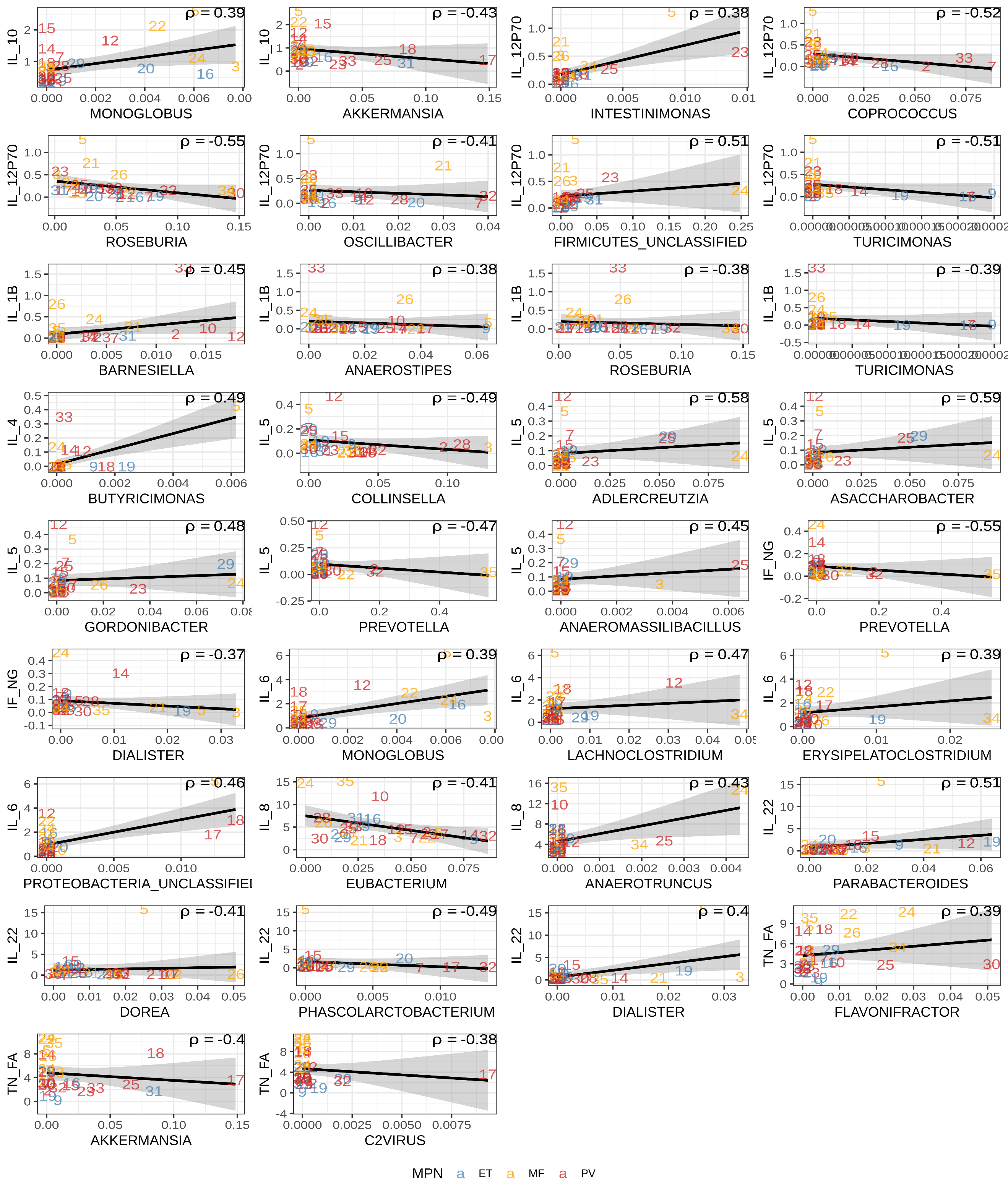


***Supplementary Figure 4:*** Scatter plots of spearman correlations between the relative abundances of microbial genera and cytokine concentrations (pg/mL). Only significant correlations are shown (p-value < 0.05). The correlation coefficient is embedded in the right of each graph. Points are labeled by the individual of origin and colored by MPN subtypes. A line represents the mean, and the shaded area delineates the 95% confidence interval.


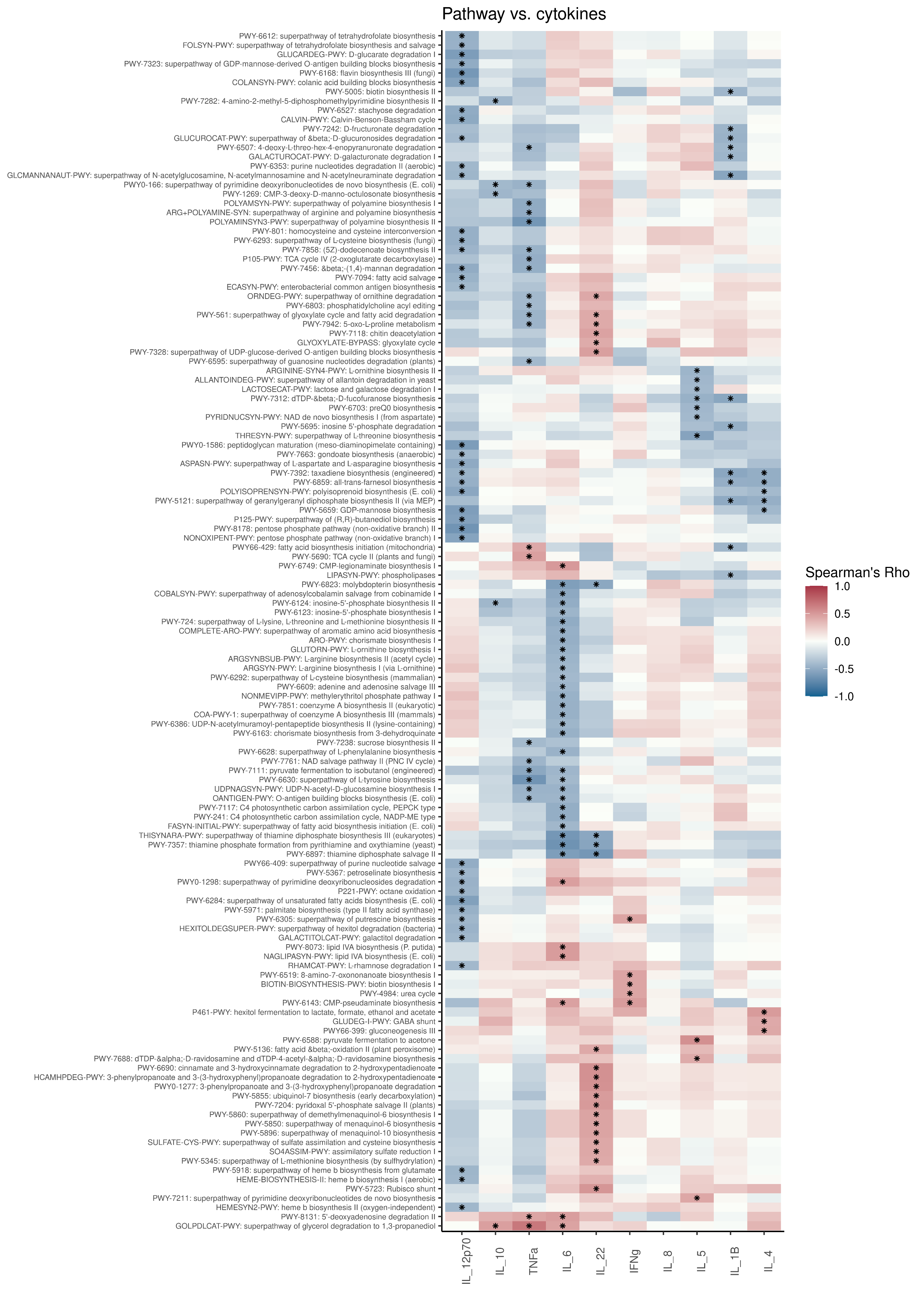


***Supplementary Figure 5:*** A heat map of metabolic pathways abundances that significantly correlated with cytokine concentrations. Asterisks denote significant correlations (p < 0.05).


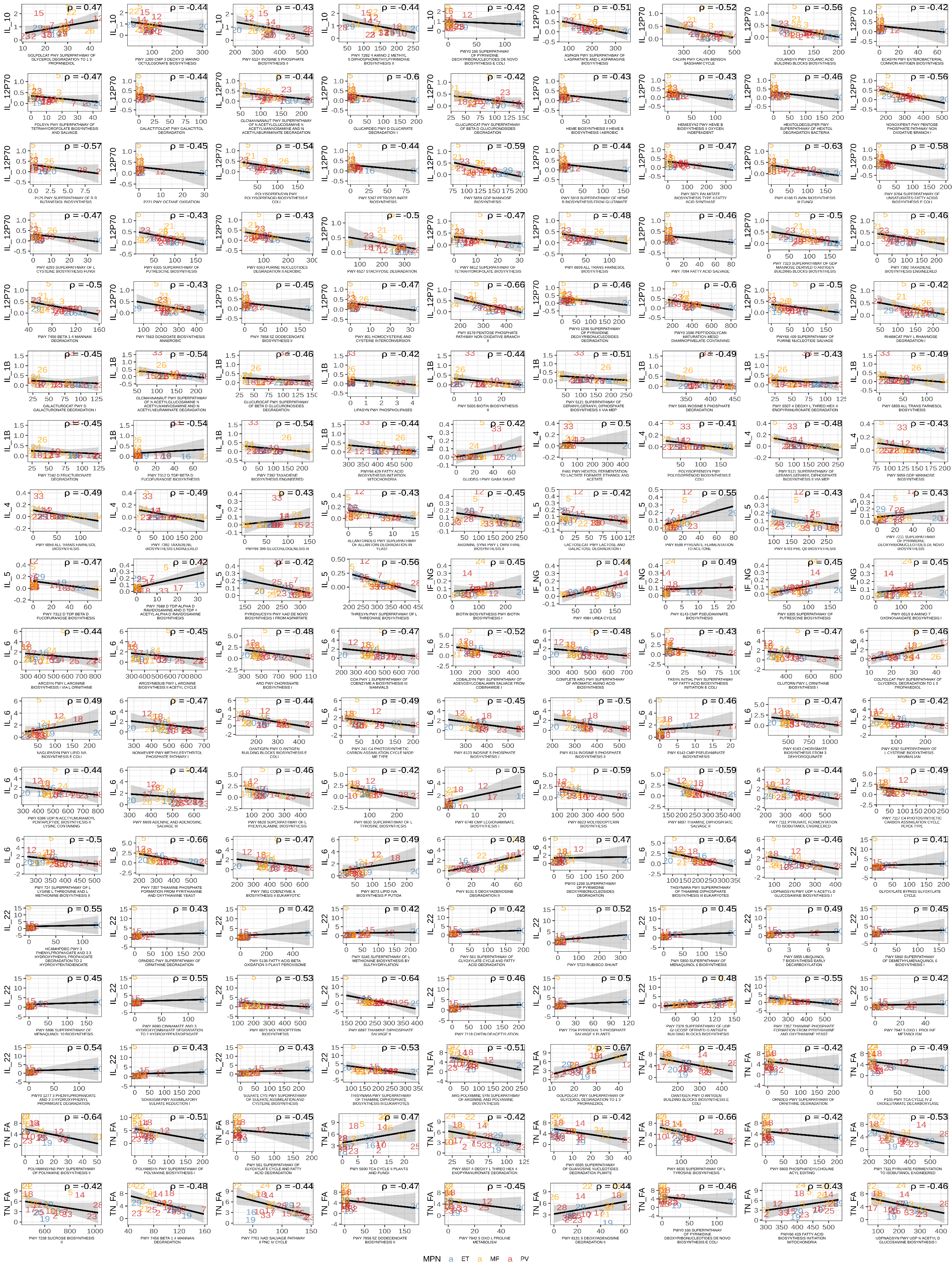


***Supplementary Figure 6:*** Scatter plots of spearman correlations between the abundances of metabolic pathways (counts per million) and cytokine concentrations (pg/mL). Only significant correlations are shown (p-value < 0.05). The correlation coefficient is embedded in the right of each graph. Points are labeled by the individual of origin and colored by MPN subtypes. A line represents the mean, and the shaded area delineates the 95% confidence interval.

**Supplementary Tables**

|  | Df | SumOfSqs | R2 | F | Pr(>F) |
| --- | --- | --- | --- | --- | --- |
| Diet | 1 | 0.410168 | 0.057337 | 1.459795 | 0.046 |
| Residual | 24 | 6.743428 | 0.942663 | NA | NA |
| Total | 25 | 7.153596 | 1 | NA | NA |

PERMANOVA formula: OTU_table[week1,] ~ Diet

***Supplementary Table 1:*** A table of results produced by PERMANOVA using only samples from week 1, demonstrating significant differences in the microbiome between diet groups pre-intervention.

|  | Df | SumOfSqs | R2 | F | Pr(>F) |
| --- | --- | --- | --- | --- | --- |
| Week | 3 | 0.199089 | 0.007196 | 0.231956 | 0.764 |
| Residual | 96 | 27.46576 | 0.992804 | NA | NA |
| Total | 99 | 27.66485 | 1 | NA | NA |

PERMANOVA formula: OTU_table ~ Week, strata = Subject

***Supplementary Table 2:*** A table of results produced by PERMANOVA, which tested whether samples across all four time points (Weeks 1, 6, 9, and 15) significantly differed in microbial composition within each individual.

| ET | Df | SumOfSqs | R2 | F | Pr(>F) |
| --- | --- | --- | --- | --- | --- |
| Week | 3 | 0.240761 | 0.045829 | 0.288184 | 0.631 |
| Residual | 18 | 5.012647 | 0.954171 | NA | NA |
| Total | 21 | 5.253408 | 1 | NA | NA |
|  |  |  |  |  |  |
| MF | Df | SumOfSqs | R2 | F | Pr(>F) |
| Week | 3 | 0.210731 | 0.026236 | 0.197581 | 0.603 |
| Residual | 22 | 7.8214 | 0.973764 | NA | NA |
| Total | 25 | 8.032131 | 1 | NA | NA |
|  |  |  |  |  |  |
| PV | Df | SumOfSqs | R2 | F | Pr(>F) |
| Week | 3 | 0.197215 | 0.01567 | 0.254711 | 0.769 |
| Residual | 48 | 12.38828 | 0.98433 | NA | NA |
| Total | 51 | 12.58549 | 1 | NA | NA |

PERMANOVA formula: OTU_table[ET/MF/PV,] ~ Week, strata = Subject

***Supplementary Table 3:*** A table of results produced by PERMANOVA, which tested whether samples across all four time points (Weeks 1, 6, 9, and 15) significantly differed in microbial composition within each individual one MPN subtype at a time.

|  | Df | SumOfSqs | R2 | F | Pr(>F) |
| --- | --- | --- | --- | --- | --- |
| Week | 3 | 0.303795 | 0.009031 | 0.291641 | 0.793 |
| Residual | 96 | 33.33357 | 0.990969 | NA | NA |
| Total | 99 | 33.63736 | 1 | NA | NA |

PERMANOVA formula: Gene_table ~ Week, strata = Subject

***Supplementary Table 4:*** A table of results produced by PERMANOVA, which tested whether samples across all four time points (Weeks 1, 6, 9, and 15) significantly differed in microbial gene composition within each individual.

|  | Df | SumOfSqs | R2 | F | Pr(>F) |
| --- | --- | --- | --- | --- | --- |
| dna_extraction | 3 | 4.0661 | 0.1470 | 23.3015 | 0.001 |
| library_prep | 1 | 0.7899 | 0.0286 | 13.5804 | 0.001 |
| Age | 1 | 0.9292 | 0.0336 | 15.9746 | 0.001 |
| Sex | 1 | 0.9196 | 0.0332 | 15.8096 | 0.001 |
| BMI | 1 | 0.6727 | 0.0243 | 11.5653 | 0.001 |
| Diet | 1 | 0.3544 | 0.0128 | 6.0927 | 0.001 |
| MPN | 2 | 1.6448 | 0.0595 | 14.1388 | 0.001 |
| MPN:Subject | 19 | 14.2166 | 0.5139 | 12.8640 | 0.001 |
| Residual | 70 | 4.0716 | 0.1472 |  |  |
| Total | 99 | 27.6648 | 1 |  |  |

PERMANOVA formula: OTU_table ~ dna_extraction + library_prep + Age + Sex + Diet + MPN / Subject, strata = Week

***Supplementary Table 5:*** A table of results produced by PERMANOVA, investigating the association of all available factors with microbial composition. Only time points from the same week were compared with each other, and subjects nested within an MPN subtype.

|  | Df | SumOfSqs | R2 | F | Pr(>F) |
| --- | --- | --- | --- | --- | --- |
| dna_extraction | 3 | 4.1883 | 0.1245 | 16.5475 | 0.001 |
| library_prep | 1 | 0.9534 | 0.0283 | 11.3003 | 0.001 |
| Age | 1 | 1.0936 | 0.0325 | 12.9622 | 0.001 |
| Sex | 1 | 1.0644 | 0.0316 | 12.6154 | 0.001 |
| BMI | 1 | 0.9241 | 0.0275 | 10.9526 | 0.001 |
| MPN | 2 | 2.2470 | 0.0668 | 13.3160 | 0.001 |
| MPN:Subject | 20 | 17.2607 | 0.5131 | 10.2291 | 0.001 |
| Residual | 70 | 5.9059 | 0.1756 |  |  |
| Total | 99 | 33.6374 | 1 |  |  |

PERMANOVA formula: Gene_table ~ dna_extraction + library_prep + Age + Sex + Diet + MPN / Subject, strata = Week

***Supplementary Table 6:*** A table of results produced by PERMANOVA, investigating the association of all available factors with microbial gene composition. Only time points from the same week were compared with each other, and subjects nested within an MPN subtype.
