# Supplementary material for: Characterizing the microbiome of patients with myeloproliferative neoplasms during a Mediterranean diet intervention
Source: mBio. 2023 Oct 25;14(6):e02308-23. doi: 10.1128/mbio.02308-23 (PMC10746218; doi:10.1128/mbio.02308-23)
Supplement: Supplemental legend — Legend for File S2. [file mbio.02308-23-s0003.docx]

***Supplementary file 2:*** This file includes the metadata and taxonomic relative abundances for each sample at the species level. The relative abundances for each sample sum to a total of one. The adherence score refers to how closely a patient adhered to a MED diet, and a score of 8/14 was considered as high adherence to a MED diet. The SAF score refers to the MPN symptom burden experienced by the individual, with a higher score reflecting a higher symptom burden. The units of measurement for cytokine concentrations are in pg/mL.
